# Appointments in the Tuberculosis Service

**Published:** 1913-10-25

**Authors:** 


### APPOINTMENTS IN THE TUBERCULOSIS SERVICE.

### Allegations of Corruption.

[This article has been sent to us by one who has had ample opportunities for observing the abuses he describes. We publish it without prejudice as an expression of individual opinion, and our columns are open to anyone who wishes to rebut the conclusions of our contributor.]

Once again has arisen the question of the relative value to those seeking election to medical appointments of professional suitability and of personal influence with the electors. It is not surprising that the matter has been brought forward in connection with the appointments which are being made as a part of those developments, following the passing into law of the Insurance Act, which are taking place in the treatment of tuberculosis. This Journal has frequently, witness the last Educational Number, emphasised the fact that the medical appointments which are being made in the Tuberculosis Service, which is still in its infancy, are of an entirely new kind; and that the Service does not possess a sound educational basis.

### The Ideal Tuberculosis Officer.

With comparative suddenness there has arisen, as the Departmental Committee on Tuberculosis foresaw there would arise, a demand for medical men to work upon the lines laid down in the Committee's reports. The personal and professional qualifications not only desirable, but essential, were broadly defined by the Committee and may be briefly recapitulated. Tuberculosis work is divided into two principal classes, which may be termed institutional and dispensary. For both, the first and all-important requirement is knowledge and experience of tuberculosis and its treatment. For institutional work training in hospital management must be combined with that special knowledge which the particular type of institution demands, whether it be a sanatorium or a hospital for cases of advanced pulmonary disease, for children or for "surgical" cases. For the other branch of the work as arranged in the Committee's scheme (the dispensary unit), the essential need is that the control be in the hands of an experienced man, with capacity for organisation, who by age, experience, and position will be acceptable as a consultant and adviser by the medical profession and the Insurance Committee.

Such were the broad definitions upon which the public authorities concerned are set the task of electing suitable officers from such candidates as may present themselves, of whose respective qualifications they have to judge. Other special, though possibly minor, considerations may have to be taken into account, as, for instance, in certain cases knowledge of locality, acquaintance with German and other medical literature, practice in *x*-ray technique, and the observation of treatment by surgical means, including the production of artificial pneumothorax. The possession of one of the higher medical degrees, the membership of the Royal College of Physicians, or a diploma in public health should obviously be taken into consideration. Whatever value may attach to any such special evidence of experience the one need is to select for appointment the candidates deemed to be, whilst personally suitable, the best educated and most highly trained for the work in hand.

### Merit or Favour?

The complaint is made, we believe not without justification, that in some instances undue regard has been paid to personal influence and considerations in favour of individual candidates to the exclusion of others better qualified for appointment —that is to say, that posts have been secured by favouritism. This is, unfortunately, by no means a new charge when made concerning the work of an elected public body. Criticism of the majority of the appointments which have been made to the post of tuberculosis officer is not suggested; many County Councils, relying upon, and trusting in, the advice and judgment of their medical officers, although sometimes hampered by a paucity of highly qualified candidates, have made an undoubtedly unbiassed selection. There are, however, other appointing bodies in which the honesty of purpose of the majority is open to doubt. It must be freely acknowledged that it is natural, that it may be right, where there is no distinction of qualification or suitability, to allow personal knowledge of a particular candidate to turn the scale in his favour. In the past posts and preferments in many, perhaps in most, of the services and professions in this country, now often gained after competitive examination, were given solely as the result of the intervention of relatives and friends.

It appears, as has been said, to be true that some of the public bodies, composed of elected representatives, allow themselves to be unfairly influenced in the election of medical men to public appointments, including, of course, those of tuberculosis medical officers. This is a serious, long-standing, and far-reaching evil, in the limitation of which publicity and plain-speaking may be of some avail. Representation leaves much to be desired. Membership of the less important "health authorities," Boards of Guardians, etc., offers, unfortunately, but little attraction to the class of man or woman best fitted to serve upon them. As a consequence of this, and of the extraordinary apathy of the electors, a class of poorly educated, unsuitable self-seekers secures nomination and only too often is returned unopposed or by the few votes necessary to overcome the feeble opposition met with at such an election. It is improbable that the small-minded and conceited members of this class of public representative often gain much financial or material benefit from their position; but, having the necessary leisure, they enjoy being in a minor way public characters, revel in the mild excitement of taking part in meetings, and feel a sense of importance in making appointments and spending public money in sums far beyond those with which they are accustomed to deal. The misfortune of the presence of such persons upon our public bodies is at the same time a cause and an effect; being there they render the position of desirable members irksome or intolerable, and so, frequently, seats are rendered vacant for occupation by more undesirables. Emphasis is laid upon this defective representation in the belief, not that the elimination of the element referred to would entirely eradicate such evils as favouritism, but that it is its presence which is conducive to them and is responsible for deterioration of authority and loss of dignity in the proceedings of a public board or council: the influence, for good or otherwise, of the clerk of such a body can hardly be overrated. The suggestion of payment of the members, possibly nearing fruition, offers but little prospect of improvement in *personnel.* Scenes are still enacted reminiscent of the meetings of the Clerkenwell Vestry of unhappy memory.

It is in the case of such minor authorities that the opportunity of appointing a medical officer is also an opportunity for the formation of cliques and for the unblushing exercise of undue influence. The members are ignorant of the value of the credentials of the applicants and unfitted to act in judgment between them. The advertisement will almost certainly have contained a clause to the effect that "canvassing either directly or indirectly will disqualify," an ineffective and practically meaningless phrase. The actual issuing of an advertisement is frequently a farce, as it by no means proves that a majority of the members has not already decided in favour of someone whose appointment is a foregone conclusion. Happy, then, is the medical man who finds himself applying before a truly honest representative body in a really open election.

The outlook in the making of public medical appointments under the minor authorities is not hopeful. Rights and privileges, pride and self-importance, the claims of blood-relationship and friendship, self-interest, gratitude, love of being on the winning side; all are factors which, since bodies of men have met together, have tended towards leadership and favouritism. It has often been necessary to make rules regulating or forbidding the appointment to paid offices of ex-members or the relatives of members. The dishonesty and self-seeking to which we have referred may hamper the selection of sanatorium officers and many everyday transactions.

### The L.G.B. as a Safeguard.

Some attempt has been made, in such instances as those we have more particularly under consideration, to cope with at least the grosser instances of unfairness. As the assent of the Local Government Board is necessary in some cases before a medical officer's appointment can be terminated by dismissal, so is the sanction of the Board required to many appointments. Useful as this provision is, its value is limited; the Board cannot undertake the duty of the local authority, and its acceptance implies less often that a candidate is suitable than that he presents no exceptional unsuitability.

There are two principles, neither entirely free from objection, upon which a selection or classification of medical men may be made: competitive examination, and submission to a tribunal of their professional brethren. Both methods are utilised in certain cases; neither is practicable in the case of, or likely to be adopted by, our minor boards and councils. Such bodies not infrequently have, however, some medical men amongst their members; upon such medical, as upon the other well-educated, members and upon the existing medical officers and the clerks falls the responsibility of using such influence as they possess to secure an honest election to every medical appointment. The "personal equation" in the case of a candidate for an appointment is one which cannot be excluded, but it becomes a different matter when it is said that personal influence is an infinitely more valuable asset to him than qualification for the work involved. In those cases in which there is lamentable failure, and loss of dignity and honesty of purpose, on the part of representatives appointed to do public work and spend public money the publicity of the press should be freely used to bring the delinquent body into the light of public opinion.

